# Neurovascular compression from a tortuous vertebrobasilar system. Technical note on surgical anatomy and cisternal microdissection

**DOI:** 10.1007/s00701-026-06945-w

**Published:** 2026-06-16

**Authors:** Zsolt Zador, Michael Lawton

**Affiliations:** 1 Abay Neuroscience Center, Wichita, KS USA; 2 Ascension Via Christi St. Francis, Wichita, KS USA; 3 Department of Neurosurgery, Barrow Neurological Institute, St. Joseph’s Hospital and Medical Center, Phoenix, AZ USA

**Keywords:** Microvascular decompression, Microdissection, Cranial base, Neuroanatomy, Microneurosurgery

## Abstract

**Background:**

Microvascular decompression (MVD) for trigeminal neuralgia (TGN) typically entails mobilisation of the superior cerebellar artery or adjacent veins. Rarely, symptomatic compression arises from a tortuous vertebrobasilar system. These cases are challenging due to eloquence and distorted microsurgical anatomy in cerebellopontine angle (CPA) often needing more complex maneuvers like vessel transposition rather than conventional interposition technique. This technical note discusses MVD technical nuances for TGN from a tortuous vertebrobasilar system.

**Method:**

Standard retrosigmoid craniotomy was carried out to access the CPA. Radioanatomical correlations within the CPA were analyzed and an exposure extending along the tortuous vertebrobasilar system was planned for surgical control and visualization. Cerebellomedullary and cerebellopontine cisterns were dissected using combination of sharp and blunt techniques to release cranial nerves and resolve neurovascular conflict.

**Conclusion:**

We demonstrate the resolution of neurovascular conflict from tortuous vertebrobasilar system using interposition technique, without needing large vessel transposition. We illustrate radioanatomical correlations within the CPA and demonstrate dissection techniques in this challenging subtype of MVDs. Our technical observations are generalizable to microneurosurgical cases.

**Supplementary Information:**

The online version contains supplementary material available at 10.1007/s00701-026-06945-w.

## Introduction

Microvascular decompression (MVD) of the trigeminal nerve typically entails the mobilisation of the superior cerebellar artery (SCA) or adjacent vein [1, 7] but in a small subset (up to 7%) of cases the symptomatic compression arises from a tortuous vertebrobasilar system [4, 8, 10]. These cases associate with complication rates up to 15% [8, 10] and are surgically challenging due to the atypical vascular configuration, multiple offending vessels, distorted CPA anatomy and the potential need for high-risk maneuvers to release the neurovascular conflict. The microneurosurgical dissection strategies required by these challenging cases are critical but these are sparsely illustrated or discussed in the literature. Clearance of the trigeminal nerve is typically achieved using a proximal to distal “sweeping” and interposition of Teflon pads described by Jannetta et al. [7]. Multiple microsurgical techniques and adjunct have been described with comparably high success rates of pain relief and these included bridge techniques [6], and padding adjunct like Teflon felt, Ivalon sponge[5], autologous muscle, adipose tissue, Gelfoam, silicone sheet, and anchoring vessels with aneurysmal clips or fascia sling [2]. These maneuvers are needed for cases where the “sweeping maneuver” with Teflon interposition is not deemed sufficient to resolve the compression. However, such maneuvers also add procedural risks with more extensive manipulation of potentially sclerosed main arteries and perforators of the vertebrobasilar system. We demonstrate a case of TGN caused by compressive tortuous vertebral artery, superior cerebellar artery (SCA) and posterior inferior cerebellar artery (PICA) branches. We illustrate the anticipated surgical anatomy and review sharp and blunt techniques for cisternal dissection. We achieve trigeminal nerve clearance of the neurovascular compression without the need for higher risk maneuvers such as sling or vessel transposition.

### Relevant surgical anatomy

**Fig. 1:**
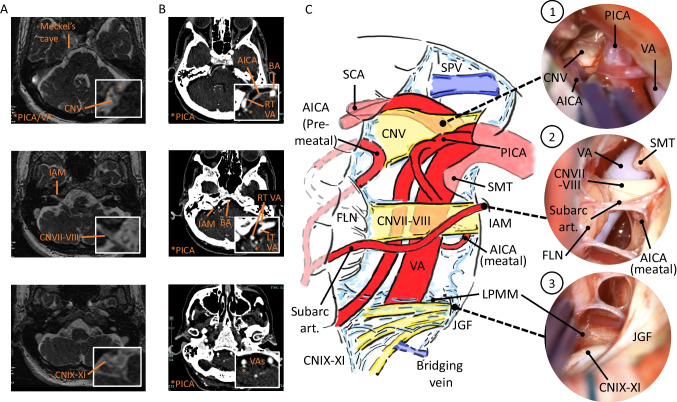
Relevant surgical anatomy. **A** and **B **Radioanatomical relations of the superior (top row), middle (middle row) and inferior (bottom row) cerebrovascular complex on preoperative high resolution T2 weighted images (**A**) with CT angiogram (**B**) sections at corresponding levels. Figure insets in A and B depict a magnified view of CNV-SCA/VA (top row), CNVII-VIII-AICA (middle row) and CNIX-XI/PICA relations (bottom row). **C** Schematic of the right sided retrosigmoid craniotomy depicting case relevant neurovascular relationships of the superior (1) middle (2) and inferior (3) neurovascular complexes. Note the relations of the tortuous VA tracking from beneath the CNIX-XI (inset 3) CNVII/CNVIII (inset 2) to the dorsal aspect of the CNV (inset 1) and the meatal AICA loop ventral to the CNVII/CNVIII giving rise to the labyrinthine artery. The latter is critical to be distinguished from the subarcuate artery in dorsal position (Inset 2) arising from the pre-meatal AICA segment)

The surgical anatomy is anticipated based on radioanatomical relations (Fig. 1). The *cerebellopontine* and *cerebellomedullary* cisterns are separated by the lateral pontomedullary membrane and typically carry the three neurovascular complexes [11]: 1) inferior complex of CNIX-XI, VA, PICA and branches, 2) middle complex CNVII-VIII, anterior inferior cerebellar artery (AICA) and branches, 3) superior complex: CNV, SCA and branches. These classic relations are altered in our case with the tortuous vertebral artery (VA) tracing anterior to the CNIX-XI, CNVII-CNVII and displacing the CNV with high PICA takeoff (Fig. 1c).

## Description of the technique

An 82-year-old female presented with 6-month duration of classic right sided trigeminal neuralgia in V2, V3 distribution. She has failed conservative management with two lines of medical therapy (carbamazepine and phenytoin). High resolution of T2 MRI sequences, MR angiogram and CT angiogram scan showed right sided tortuous vertebral artery looping up to the right cerebellopontine and cerebellomedullary cistern displacing the trigeminal nerve (Fig. 1). The patient was consented for right sided retrosigmoid craniotomy and microvascular decompression of the trigeminal nerve.

### Assessment of case specific difficulties based on radioanatomical relationships

From the CTA and high-resolution preoperative MRI of the skull base we anticipated distorted anatomy due to: 1) tortuous vertebral artery, 2) high takeoff for posterior inferior cerebellar artery both compressive and dorsal to the CNV (Fig. 1a-c) 3) anticipated perforators of tracing to the brainstem and 4) potentially atypical course of AICA branches such as subarcuate and labyrinthine artery (Fig. 1a-c), of particular relevance was that AICA tends to give of a higher number of short perforators restricting vessel transposition [9]. Further case specific considerations were the patients age and ability to tolerate prolonged surgery and the atherosclerotic, rigid vertebral artery that may limit the extent of the vessel’s mobilization. Our strategy therefore included a wide exposure of the cerebellopontine angle by dissecting cerebellomedullary, cerebellopontine cisterns, opening of the petrosal fissure with the benefit of brain relaxation, gravity retraction and exposure of atypical anatomy.

### Positioning and craniotomy

Patient was positioned in standard “park bench” and right sided retrosigmoid craniotomy carried out. Durotomy was brought up to the edge of the transverse and sigmoid sinus. Cerebellomedullary and cerebellopontine cisterns were visualized through elevation of cerebellum. We first illustrate sharp and blunt cisternal dissection techniques, then describe their stepwise implementation.

### General principles of dissection techniques

#### Sharp dissection

**Fig. 2:**
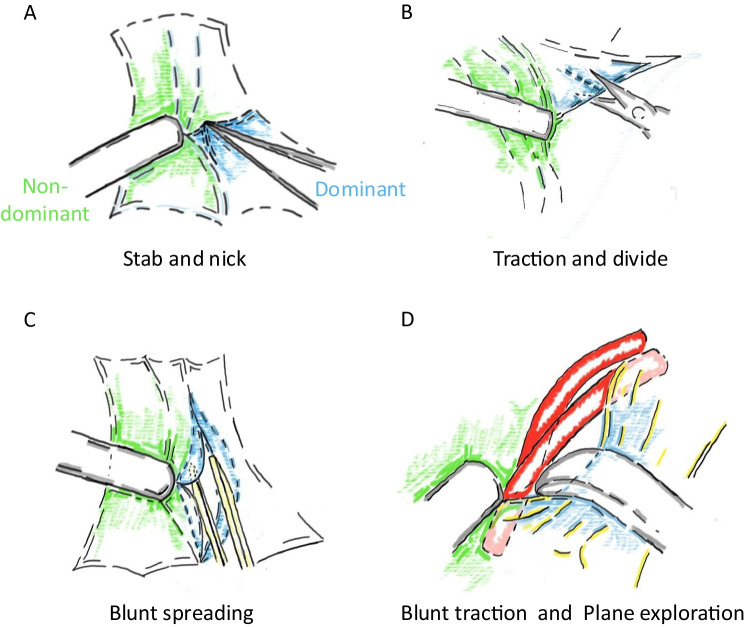
Review of sharp (**A** and **B**) and blunt microdissection (**C** and **D**) techniques. Suction is held in the non-dominant hand and places the target plane under traction for controlled sharp opening (**A**), division (**B**), blunt spreading (**C**) or plane exploration (**D**). Instrument traction footprint on the tissues is color coded green for retraction with the non-dominant hand and blue for the dominant hand

**Fig. 3:**
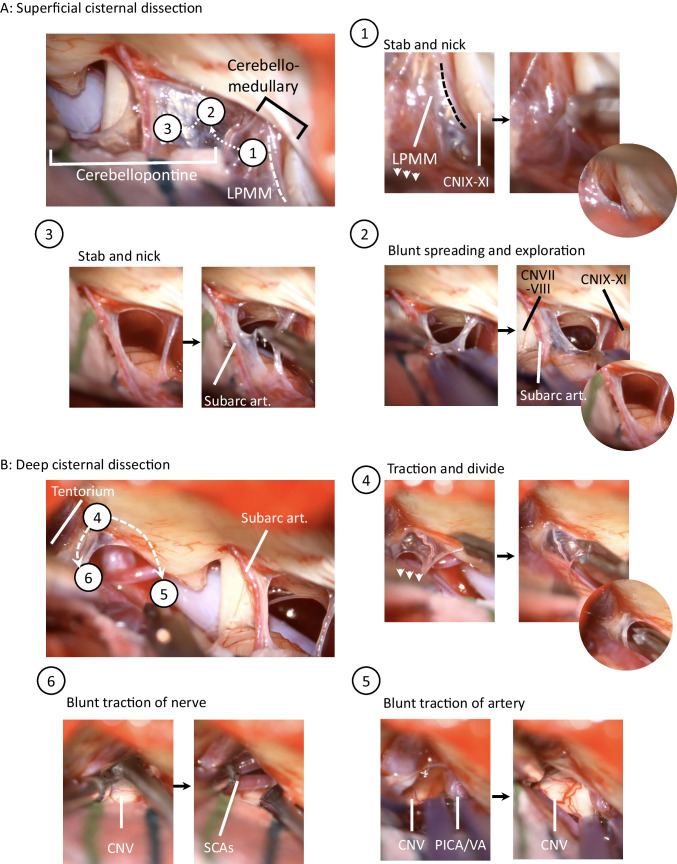
Case based illustration of microneurosurgical techniques for cisternal dissection. Superficial (**A**) and deep (**B**) cisternal dissection of the right sided cerebellomedullary and cerebellopontine cisterns. Techniques “stab and nick” (1), (3) or “traction and divide” (4) are used to open the resilient external arachnoid membrane followed by blunt spreading (2) or traction (5), (6) to dissect cisternal content (arrowheads in (1) and (4): suction traction by non-dominant hand). Circular insets in (1), (2) and (4) show anatomical result after completion of microneurosurgical dissection. Interrupted line in (A) and (1) indicates site of arachnoid opening

“Stab and nick”: the walls of the arachnoid cistern are robust and anchored. Excess traction can injure cranial nerves, tear pial vessels causing bleeding that obscures view. Sharp opening with the “stab and nick” maneuver minimizes this risk (Fig. 2a and Fig. 3a, inset 1). The plane of the dorsal arachnoid cistern walls in CPA are perpendicular to our line of sight. This membrane is elevated using suction in non-dominant hand and oval opening is created using arachnoid knife.

“Traction and divide”: The free edge of the arachnoid opening can be extended sharply with microscissors or arachnoid blade (Fig. 2b and Fig. 3b, inset 4). Traction with microsuction in the non-dominant hand brings the arachnoid into a fold and the microscissors/arachnoid knife in the dominant hand is engaged to divide in the edge.

#### Blunt dissection

“Blunt spreading”: sharply opened planes can be mobilized using microforceps/bipolar forceps with engaging the closed forceps tips in between the target planes and allowing the passive opening force of instrument to spread (Fig. 2c and Fig. 3a, inset 2). This maneuver divides arachnoid trabeculae within a cistern, extends opening in the cisternal wall and separate adjacent neurovascular structures.

“Bunt traction”: structures can be mobilized gently using suction, forceps or microdissector to free arachnoid adhesions (Fig. 2d and Fig. 3b, insets 5 and 6). With the microsuction in non-dominant hand the target plane is held under traction and parallel plane is developed with blunt dissector. The dissection trajectory runs in parallel to the plane of the target structure.

“Plane exploration”: used when boundaries between target planes are not clear or content of arachnoid fold is obscured (Fig. 2d and Fig. 3a, inset 2). Combination of blunt spreading and parallel dissection can be used to explore, visualize structures. It involves tilting to different angles and distinguish adjacent structures based on how they move in relation to one another.

### Case specific implementation of dissection techniques (OPERATIVE VIDEO)

We sought to expose the entire length of the VA/PICA complex 1) due to its anticipated proximity the superior, middle and inferior cranial nerve complexes 2) to visualize anticipated variants of perforators and critical branches (labyrinthine and subarcuate artery, short/restricting AICA branches [9]). The *cerebellomedullary cistern* was chosen for initial exposure because of shallow access available after sufficient occipital bone removal granting early CSF release and brain relaxation. The dorsal membrane to *cerebellomedullary cistern* is opened sharply, accessing the inferior neurovascular complex (Fig. 3a, inset 3) along with the lateral pontomedullary membrane (LPMM). Inferior bridging vein dorsal to the CN IX-XI complex can be divided to better mobilise the cerebellum. The PICA enters deep in the surgical view from below the rootlets of the glossopharyngeal nerve or rarely (4%) loops above the nerve [11]. PICA distal to CNIX-XI is often covered by the flocculonodular lobule (“tonsillomedullary” segment), rarely needs dissection in MVDs. In our case PICA has a high take-off due to the VA loop adjacent to the CNV. Consequently, it is encountered in the upper neurovascular domain. We then release strands of the lateral pontomedullary membrane as its draped across the CNIX-XI and entered the cerebellopontine cistern (Fig. 3a, inset 3).

**Fig. 4:**
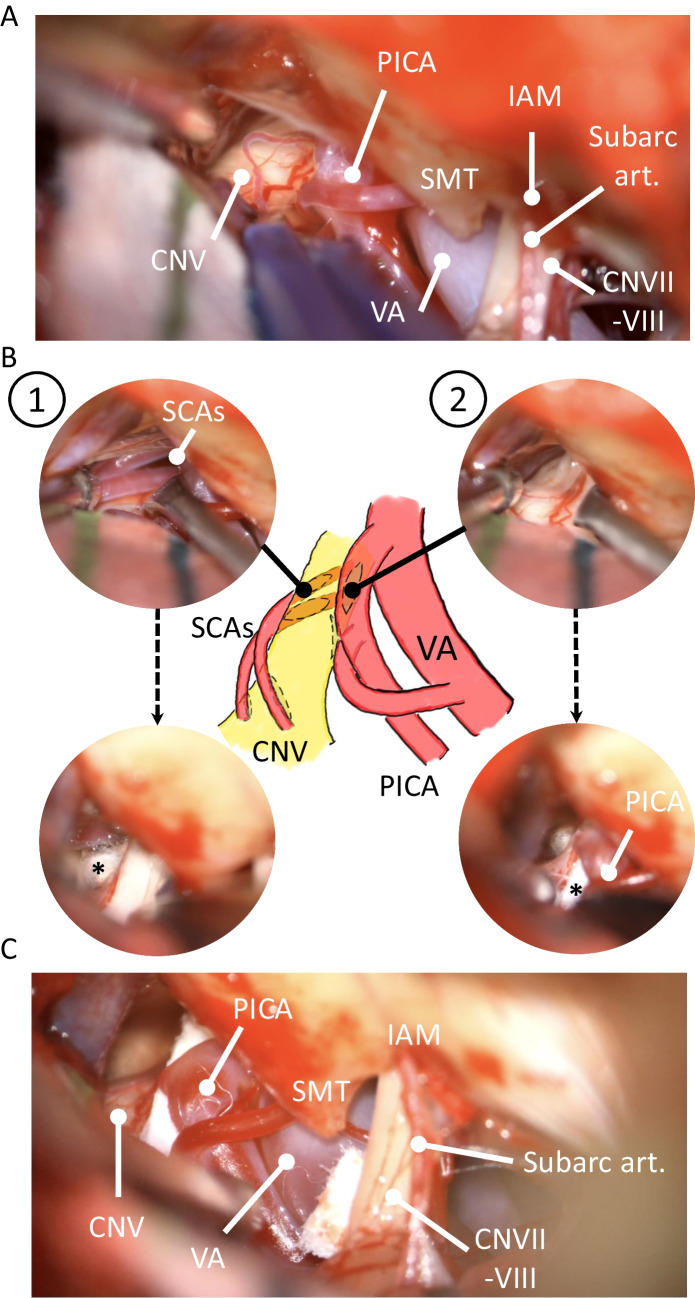
Trigeminal nerve dissection in the right CPA. Surgical view of the fully dissected cerebellopontine and cerebellomedullary cisterns. Note the compressive VA/PICA complex overlying the CNV and VA tracking from under the CNVII-CNVIII bundle. **B **Trigeminal nerve dissection: center: schematic depicting the neurovascular conflict with shaded areas shown in **A**. Inset 1: Surgical view of exploring the superior aspect of the nerve, separating and padding of the SCA/CNV conflict with Teflon (asterisk). Inset 2: Surgical view depicting the separation of the caudal neurovascular conflict with blunt retraction of the caudal neurovascular conflict from the PICA/VA complex with interposed Teflon pads (asterisk). C: Surgical view after completion of MVD with Teflon pads in situ. Patient was pain free at 1 year follow-up

The *cerebellopontine cistern* carries the superior and middle neurovascular complex. At its center CNVII-VIII with adjacent AICA loops, labyrinthine and subarcuate arteries (Fig. 1c, 3b, 4a) constitute the *middle neurovascular complex*. Limited opening was made in the horizontal fissure to release the floculonodular lobe and minimize manipulation of the subarcuate artery and CNVII-VIII complex. The subarcuate artery is superficial to the CNVII-VIII complex and is encountered first. It courses towards the temporal bone’s subarcuate fossa after coming off the “post-meatal” AICA segment (Fig. 1c, 3b, 4a and c). The labyrinthine artery lies deeper, in front of the CNVII-CNVIII complex arising from the more proximal “meatal” AICA segment (Fig. 1C, Inset 2). This non-compressive AICA segment is briefly visualized, left undisturbed minimizing injury risk and hearing compromise. Most superficial/superior in the surgical field is the tentorium with superior petrosal vein(s). Latter can be taken as required (Fig. 1c). The *superior neurovascular complex* comes into view as we focus deeper into the space between the tentorium and the CNVII-VIII. Compressive neurovascular relations of CNV are the SCA loops superiorly (Fig. 3b, inset 6, Fig. 4b inset 1) and VA/PICA complex dorsally/inferiorly (Fig. 3b, inset 5, Fig. 4b inset 2).

#### Trigeminal nerve dissection

The surgical corridor to the CNV was narrowed by a dominant suprameatal tubercule (Fig. 4a) and division of the superior petrosal vein was considered to expand access. However sharp release of the anchoring arachnoid of the CNV was sufficient for visualize the nerve and its neurovascular conflicts. The relations of the CNV were explored with blunt dissection (without translating traction onto the neighbouring neurovascular structures (Fig. 4b). Inferior aspect of the CNV is gently mobilized to expose the compressive VA/PICA complex (Fig. 4b, inset 2). Caudal corner of the CNV nerve root entry zone was visualized and cleared. We then explored the superior and ventral aspects (Fig. 4b, inset 1) of CNV had duplicated SCAs draped over the CNV, and mobilised using parallel dissection clearing all neurovascular conflict (Fig. 4b, inset 1 and 2).

#### Teflon padding

Teflon pads were positioned around CNV by sliding the pads along the superior/inferior aspects of the nerve root entry zone and advanced proximal to distal. This creates a Teflon “sandwich”, that pads the nerve from the SCA, PICA/VA complex (Fig. 4b and c).

#### Clinical course and outcome

Patient postoperative course was uneventful, she was discharged home on postop day 3 and remained pain free at 1 year follow-up.

### Indications

TGN is primarily a clinical diagnosis: paroxysmal pain with classic triggers (chewing, speaking, shaving or washing the face). Dental/Neurologist review are important for alternative causes like dental problems, trigeminal neuropathy, multiple sclerosis, temporomandibular joint disorder or chronic sinusitis. MVD is indicated when medical management with sodium channel blockers (carbamazepine/oxacarbamazepine) fails.

## Limitations

MVD has success rates of 80–95% at 3 years and revision surgery or neurotomy procedures are an option [1]. Postoperative hearing compromise and trigeminal nerve dysfunction are rare. With drastic nerve compression additional maneuvers may be considered: bridge techniques, Teflon felt, Ivalon sponge, autologous muscle, adipose tissue, Gelfoam, silicone sheet, and retraction assisted with aneurysmal clips [4, 12]. However, these maneuvers are needed for cases where the “sweeping maneuver” and Teflon padding is not deemed sufficient, they do however add procedural risks by virtue of more extensive manipulation of the vertebrobasilar system. In cases of severe atherosclerosis repositioning may cause lumen compromise and devastating stroke. Perforators arising from the vertebrobasilar system may also be a limitation to the extent of vessel mobilization.

## How to avoid complications

Optimal patient position, exposure of the transvers sigmoid edge, wide arachnoid dissection are important for surgical visualization and to preserve high risk neurovascular relations (CNV neurovascular conflict, labyrinthine/subarcuate artery, bridging veins). Particularly short perforators arising from the offending vessel have been documented to limit decompression up to half the cases [9]. Use of neuromonitoring/cranial nerve monitoring, brainstem auditory evoked potentials can serve as an early warning of compromise. Intraoperative ICG, angiogram or doppler can help identify vascular compromise from surgical dissection or transposition of the conflicting vessels.

## Specific information for the patient

The standard craniotomy risks, specific to the procedure are risk of hearing loss, facial numbness and recurrence of the pain. Hospital stay is typically 2–3 days and pain relief is often immediate although may be delayed by several months [3]. TGN is primarily a clinical diagnosis and pain relief may unmask other problems such as dental or TMJ pathology.

## Key points of summary

- preoperative MRI and CTA to anticipate surgical anatomy
- positioning is important to optimize surgical visualization
- wide exposure of the arachnoid membrane to establish the atypical anatomy
- Superior petrosal and inferior petrosal bridging veins can be divided if access requires
- Use sharp arachnoid dissection for outer membrane to avoid traction on vessels
- Arachnoid is elevated with the non-dominant hand and divided sharply with scissors or arachnoid knife in dominant hand
- proximal to distal dissection of the CNV works well in most cases to free up the neurovascular conflicts
- There are likely multiple compressive vessels denting the CNV
- “Sweeping” technique is safe and should be the first port of call. For drastic CNV compressions consider more aggressive, higher risk options (sling, vessel transposition)
- use intraoperative neuromonitoring as early warning of CN or brain stem compromise

## Supplementary Information

Below is the link to the electronic supplementary material.

Supplementary file1 (MP4 459756 KB)

## Data Availability

No datasets were generated or analysed during the current study.

## Abbreviations

MVD: Microvascular decompression
SCA: Superior cerebellar artery
AICA: Anterior inferior cerebellar artery
PICA: Posterior inferior cerebellar artery
CNV: Trigeminal nerve
CNVII: Facial nerve
CNVIII: Vestibulocochlear nerve
CNIX: Glossopharyngeal nerve
CNX: Vagus nerve
CNXI: Accessory nerve
LPMM: Lateral pontomedullary membrane
TGN: Trigeminal neuralgia
CPA: Cerebellopontine angle
SMT: Suprameatal tubercle
TMJ: Temporomandibular joint
JGF: Jugular foramen
FLN: Flocculonodular lobe
Subarc art.: Subarcuate artery
SPV: Superior petrosal vein
